# Establishing Criteria for Human Mesenchymal Stem Cell Potency

**DOI:** 10.1002/stem.1982

**Published:** 2015-05-21

**Authors:** Rebekah M. Samsonraj, Bina Rai, Padmapriya Sathiyanathan, Kia Joo Puan, Olaf Rötzschke, James H. Hui, Michael Raghunath, Lawrence W. Stanton, Victor Nurcombe, Simon M. Cool

**Affiliations:** ^1^ Glycotherapeutics Group; ^2^ Advanced Wound Care LaboratoryInstitute of Medical Biology, A*STARSingapore; ^3^ Department of Biomedical Engineering; ^4^ Department of Biochemistry; ^5^ NUS Tissue Engineering Programme; ^6^ Department of Orthopedic SurgeryNational University of SingaporeSingapore; ^7^ Department of Biological SciencesNational University of SingaporeSingapore; ^8^ Singapore Immunology Network (SIgN), A*STARSingapore; ^9^ Stem Cell and Regenerative BiologyGenome Institute of Singapore, A*STARSingapore; ^10^ School of Biological SciencesNanyang Technological UniversitySingapore; ^11^ Lee Kong Chian School of Medicine, Nanyang Technological UniversitySingapore; ^12^ Sciences, Singapore University of Technology and Design8 Somapah RoadSingapore

**Keywords:** Mesenchymal stem/stromal cells, Quality, Growth capacity, Potency, STRO‐1, PDGFR‐α

## Abstract

This study sought to identify critical determinants of mesenchymal stem cell (MSC) potency using in vitro and in vivo attributes of cells isolated from the bone marrow of age‐ and sex‐matched donors. Adherence to plastic was not indicative of potency, yet capacity for long‐term expansion in vitro varied considerably between donors, allowing the grouping of MSCs from the donors into either those with high‐growth capacity or low‐growth capacity. Using this grouping strategy, high‐growth capacity MSCs were smaller in size, had greater colony‐forming efficiency, and had longer telomeres. Cell‐surface biomarker analysis revealed that the International Society for Cellular Therapy (ISCT) criteria did not distinguish between high‐growth capacity and low‐growth capacity MSCs, whereas STRO‐1 and platelet‐derived growth factor receptor alpha were preferentially expressed on high‐growth capacity MSCs. These cells also had the highest mean expression of the mRNA transcripts *TWIST‐1* and *DERMO‐1*. Irrespective of these differences, both groups of donor MSCs produced similar levels of key growth factors and cytokines involved in tissue regeneration and were capable of multilineage differentiation. However, high‐growth capacity MSCs produced approximately double the volume of mineralized tissue compared to low‐growth capacity MSCs when assessed for ectopic bone‐forming ability. The additional phenotypic criteria presented in this study when combined with the existing ISCT minimum criteria and working proposal will permit an improved assessment of MSC potency and provide a basis for establishing the quality of MSCs prior to their therapeutic application. Stem Cells *2015;33:1878–1891*

## Introduction

Potency assays that predict the therapeutic efficacy of bone marrow‐derived adult mesenchymal stem cells (MSCs) are critical to their successful application in regenerative medicine. Patients are still currently receiving cells of unproven potency, which in turn, may contribute to many of the suboptimal outcomes that are being reported 1. Even in cases where MSC therapy has proven beneficial, use of cells with higher potency may result in improved efficacy.

However, despite a considerable body of research over the last decade, resulting in an increasing pace of investigational new drug (IND) submissions to the U.S. FDA, there is still no generally accepted definition of what constitutes a mesenchymal stem (or stromal) cell 2, 3. In view of the growing clinical utility of MSCs, there is a pressing need to develop better standards that more accurately establish their potency. These standards should be broadened beyond those originally nominated by the International Society for Cellular Therapy (ISCT) 4 that are proving insufficient predictors of therapeutic success, even though this may not have been their intended use. For example, other notable criteria that include paracrine factors 5 as well as immunomodulatory status 6, 7 need to be considered. The need for better reference standards for MSCs was also recently emphasized by Prockop et al. 8.

The problem in establishing such benchmarks is that MSCs consist largely of heterogeneous subpopulations of adherent cells that are typically isolated from adult bone marrow that, when induced, are capable of self‐renewal, colony formation, and multilineage differentiation 9, 10, 11, 12. However, the ability of MSCs to survive and differentiate in vitro has proven a poor predictor of their ability to repair tissue 13, 14, 15. Efforts to increase MSC potency by deriving populations from single cells have failed to increase the proportion of MSCs in the subsequent population of passaged cells 16. Also, strategies for enriching MSCs exploiting one or more cell‐surface markers have gained favor, particularly for markers such as stromal precursor antigen‐1 (STRO‐1) 17, 18, SSEA‐4 19, CD49a 20, CD271 21, CD146 22, leptin receptor 23, and the use of size‐sorting from adherent cultures of bone marrow aspirates 11, 24. Yet these enriched MSCs still require extensive expansion prior to reimplantation or banking that may result in a subsequent increase in cell heterogeneity and a presumptive decrease in potency.

Another characteristic of MSCs gaining attention is the trophic factors they produce 5, 25. Vascular endothelial growth factor (VEGF) has been shown to play crucial roles in the repair of heart failure 26, hepatocyte growth factor (HGF) in the amelioration of multiple sclerosis 27 and graft‐versus‐host disease, and insulin‐like growth factor (IGF‐1) and epidermal growth factor (EGF) in wound healing 28. These factors are thought to play critical roles in cell engraftment and wound healing. Moreover, it may be possible to link MSC potency directly to their secretory profile 29, 30.

Here, we sought to identify critical determinants of MSC potency by linking in vitro attributes such as self‐renewal and clonogenicity, cell size, telomere length, mRNA profile, and growth factor/cytokine secretion to in vivo tissue regeneration ability as determined by their ectopic bone‐forming ability. This study highlights that the minimum criteria suggested by the ISCT for defining MSCs are not in themselves predictive of stem cell potency. There is thus a need for common guidelines and protocols for the characterization of MSCs for clinical use, as suggested in the ISCT working proposal 31. Therefore, in an effort to improve MSC characterization, our data suggest the need to include additional criteria when assessing MSC functional potency which include the rate of self‐renewal, expression of the mRNA transcripts *TWIST‐1* and *DERMO‐1*, and the expression of the cell‐surface biomarkers STRO‐1 and platelet‐derived growth factor receptor alpha (PDGFR‐α).

## Materials and Methods

### Isolation and Culture of MSCs

Human MSCs were isolated from the bone marrow of six healthy donors with no reported antibiotic sensitivity (Supporting Information Table S1) by plating human bone marrow mononuclear cell (MNC) fractions (Lonza, Walkersville, MD, www.lonza.com) in maintenance media comprising of Dulbecco's Modified Eagle's medium (DMEM) (DMEM‐low glucose, 1,000 mg/l) with 10% fetal calf serum (Hyclone, Logan, UT, www.hyclone.com), 2 mM l‐glutamine, 50 U/ml penicillin, and 50 U/ml streptomycin (Sigma‐Aldrich, St. Louis, MO, www.sigmaaldrich.com), as described previously 32. Briefly, mononuclear cells were washed in maintenance media, centrifuged, resuspended in media, and plated at a density of 50,000 cells per square centimeter in T175 flasks. Cells were allowed to adhere for 7 days following which nonadherent cells were removed and fresh media added. The plastic adherent cells were cultured for 3 weeks, harvested, and counted (Supporting Information Fig. S1B) before replating them for expansion at a density of 5,000 cells per square centimeter. Cells from passage 4 were used for all experiments, unless otherwise stated. Individual donor MSCs were characterized and experimented on as separate MSC populations throughout, and there was no parameter upfront that distinguished the plated cells of different donors. For colony‐forming units‐fibroblastic (CFU‐F) assays, MNCs were plated at 0.5–3.0 × 10^6^ cells in T75 flasks, allowed to adhere and form colonies, and stained with 0.5% crystal violet (Sigma‐Aldrich) after 14 days. Visible colonies were quantified only when they were greater than or equal to 50 cells, and not in contact with an adjacent colony. Efficiency was calculated by estimating the number of colonies formed per 10^5^ cells plated.

### Cell‐Size Analysis

Single‐cell suspensions of MSCs were stained with Annexin V, washed, and resuspended in MACS buffer (2 mM EDTA, 0.5% bovine serum albumin (BSA) in PBS, pH 7.2) before analysis using FACSArray Bioanalyzer (BD Biosciences, San Jose, CA, www.bdbiosciences.com). After gating out Annexin V^+^ cells, a quadrant gate was applied to the live population, and the fraction of low forward scatter/side scatter (FSC/SSC) events recorded to obtain the relative percentage of small‐sized cells.

### Cumulative Growth and Telomere Length Analysis

To assess MSC growth, cells were seeded at 5,000 cells per square centimeter and cultured under maintenance conditions. At 70%–80% confluence, cells were enzymatically removed with 0.125% trypsin/Versene and viable cells counted using Guava EasyCyte Plus (Milipore, Merck KGaA, Darmstadt, Germany, www.merckmillipore.com). Cells were replated at the same density and progressively subcultured for 13–15 passages (i.e., until senescence) to estimate cumulative cell numbers and population doublings. Genomic DNA at different passages was isolated and telomere length analysis performed as described previously 33.

### Flow Cytometry

Single‐cell suspensions were stained with phycoerythrin‐ or fluorescein isothiocyanate‐conjugated anti‐human CD105 (266), CD73 (AD2), CD90 (5E10), CD45 (H130), CD34 (RAM34), CD49a (SR84), CD29 (MAR4), EGF‐R (EGFR.1), IGF‐IRα (CD221, 1H7), NGF‐R (CD271, C40–1457), PDGFR‐α and β (CD140a, αR1 and CD140b, 28D4), CD11b (D12), HLA‐DR (TU36), CD19 (HIB19), CD14 (MфP9), CD106 (51‐10C9), CD146 (P1H12), SSEA‐4 (MC813‐70), STRO‐1 antibodies or the isotype‐matched controls IgG1κ (MOPC‐21), IgG2aκ (G155‐178), IgG2bκ (27−35), IgM, and IgG3 (A112‐3), and analyzed on a BD FACSArray Bioanalyzer and FlowJo software v8.0 (Tree Star, Inc. Ashland, OR, http://www.flowjo.com) by gating at 2% of isotype control. For each marker and isotype, 20,000 events were acquired. All antibodies were purchased from BD Biosciences except IgM (μ‐chain specific) (Caltag laboratories, Burlingame, CA, www.lifetechnologies.com) and STRO‐1 (kindly provided by Prof. Stan Gronthos, School of Medical Sciences, Faculty of Health Sciences, University of Adelaide, Australia).

### Immunomodulation Assay

All experiments using human blood were approved by the Institutional Review Board at the National University of Singapore (NUS‐IRB 09‐256) and comply with the Helsinki declaration. Written informed consent was obtained from all blood donors prior to blood collection. Adult human blood was collected in BD K2 EDTA Vacutainers (Becton Dickinson, Franklin Lakes, NJ, www.bd.com) and PBMCs were isolated by Ficoll‐Paque PLUS (GE Healthcare, Freiburg, Germany, www.gelifesciences.com) density gradient centrifugation (400 x g for 30 min without brake). CD4^+^ T cells were isolated from PBMCs using EasySep negative selection human CD4^+^ T cell enrichment kit (Stem Cell Technologies, Vancouver, BC, http://www.stemcell.com) and labeled with 5 µM carboxy‐fluorescein diacetate succinimidyl ester (CFDA‐SE kit, Life Technologies, Molecular Probes, Carlsbad, CA, www.lifetechnologies.com). CD4^+^ CFSE^+^ cells (0.5–1 × 10^5^) were stimulated by anti‐CD3/anti‐CD28 MACSiBeads (Miltenyi Biotec, Bergisch Gladbach, Germany, www.miltenyibiotec.com) at a ratio of four beads per T cell, to which MSCs were added at different T cell/MSC ratios and incubated at 37°C. Proliferation of CD4^+^ CFSE^+^ cells was measured after 7 days by flow cytometry and the percentage inhibition of T‐cell proliferation determined.

### Multiplex Detection of Growth Factors and Cytokines

Cells were cultured in maintenance media for 4 days and conditioned media collected thereafter. To release matrix bound proteins, cell layers were washed with 2 M NaCl in 20 mM HEPES (pH 7.4) for ∼10 seconds. The conditioned media and salt wash were analyzed separately using Human Cytokine/Chemokine kit (MPXHCYTO‐60K, Millipore) for simultaneous quantification of 14 growth factors and cytokines, according to the manufacturer's instructions. Results are presented as logarithmic concentration for each analyte (growth factor or cytokine), which includes factors bound to matrix as well as secreted into media. Results are shown as mean of two individual experiments for each donor.

### Gene Expression Analysis

For quantitative PCR, total RNA was isolated using a Nucleospin RNA II kit (Macherey Nagel, Bethlehem, PA, www.mn-net.com) according to the manufacturer's instructions and quantified by NanoDrop 1000 (Thermo Fisher Scientific, Inc. Wilmington, DE, www.thermofisher.com). After conversion to cDNA (Superscript VILO, Life Technologies, Invitrogen, Carlsbad, CA, www.lifetechnologies.com), expression of mesodermal genes *TWIST‐1* and *DERMO‐1*, osteogenic markers *RUNX2*, *ALP*, and *BSP‐II*, adipogenic markers *PPARγ* and *CEBPα*, and chondrogenic genes *COL2A1* and *SOX9* was assessed using TaqMan Gene Expression assays on an Applied Biosystems 7500 PCR System (Life Technologies, Applied Biosystems, Carlsbad, CA, www.lifetechnologies.com) (Supporting Information Table S2). *C*_T_ values were normalized to *β‐ACTIN* and results plotted as relative expression units.

For microarray, RNA extracted from MSCs was amplified using a TotalPrep RNA amplification kit according to the manufacturer's instructions (Life Technologies, Ambion, Grand Island, NY, www.lifetechnologies.com). The resulting purified biotin‐labeled complementary RNA (cRNA) was normalized and hybridized onto a HumanHT‐12 version 4 beadchip (Illumina, San Diego, CA, http://www.illumina.com) using direct hybridization. The chip was then washed, blocked, and Cy3‐streptavidin bound to the hybridized cRNA. An Illumina BeadArray Reader using the Illumina BeadScan software was used to image the chip, and the image data converted into an expression profile by GenomeStudio (Illumina). After background subtraction, data were submitted to GeneSpring (Agilient Technologies, Santa Clara, CA, www.agilent.com). The replicates were averaged and pairwise analysis performed, followed by a Student's *t* test with *p* < .05 and fold change ≥1.5. Two donor samples representing each group with two technical replicates were analyzed. The gene lists generated were uploaded using Entrez gene ID onto DAVID (david.abcc.ncifcrf.gov) for functional annotation clustering by GOTERM_BP_FAT with medium classification stringency 34. Only biological processes with *p* ≤ .05 were considered.

### Multilineage Differentiation

Evaluation of the differentiation potential of MSCs for osteogenic, adipogenic, and chondrogenic lineages was performed as described previously 32. Average intensities of stained wells/pellets were quantified using Quantity One software (Bio‐Rad Laboratories, Hercules, CA, www.bio-rad.com). For the von Kossa and oil red O methods, the staining intensity values were normalized to DNA content. Results are expressed as relative intensity per microgram DNA (fold‐increases of intensity in treatment wells compared to their respective control wells; Supporting Information Fig. S4). In these measurements, a darker stain implies a higher density value.

### Ectopic Bone Formation Assay

Comparison of in vivo efficacy between individual MSC donors was assessed by ectopic bone formation assay. The experimental groups included MASTERGRAFT Matrix (Medtronic, Minneapolis, MN, www.medtronic.com) alone (control) and MASTERGRAFT Matrix with MSC from 6 individual donors (donors A, B, C, D, E, and F) resulting in seven groups with 5 implants per group (35 implants in total). The implants were randomly assigned to 18 mice, with up to 2 implants per animal. Donor MSCs at P4 (3 × 10^6^ cells) were seeded onto scaffolds before implantation into subcutaneous pockets of 8‐week‐old immunodeficient mice (NIH‐bg‐nu‐xid, Harlan Sprague‐Dawley, Harlan Laboratories, Indianapolis, IN, www.harlan.com), as described previously 35. Surgeries were performed according to the specifications of an ethics‐approved small animal protocol (IACUC: #110651). An incision of 1 cm was created dorsally on either side of the vertebral column and a subcutaneous pocket created by blunt dissection. One implant was placed in each pocket and incisions closed with surgical staples. X‐rays were taken immediately postimplantation and again at 8 weeks using a Shimadzu MobileArt MUX‐101 Standard (Shimadzu MobileArt MUX‐101 Standard, Tokyo, Japan, www.shimadzu.com) and a DÜRR MEDICAL‐CR 35 VET Image Plate Scanner DÜRR MEDICAL‐CR 35 VET Image Plate Scanner, Vancouver, WA, www.im3vet.com). At sacrifice (8 weeks), implants were removed, digitally photographed (Nikon D80), and imaged using a Skyscan 1076 µCT scanner (Skyscan, Kontich, Belgium, www.skyscan.be/) under parameters previously reported 36. Reconstructed images (three‐dimensional) and bone volume measurements were performed using Mimics 14.0 software (Materialise, Leuven, Belgium, www.materialise.com) after applying appropriate threshold settings. After µCT analysis, implants were transferred to 10% neutral buffered formalin under vacuum for 1 week, decalcified, paraffin embedded, sectioned, and stained as previously reported 36, 37. Immunohistochemistry was also performed using primary antibodies to mouse osteocalcin (M188, 1:100, Takara Bio, Shiga, Japan, www.takara-bio.com) and human osteocalcin (ab76690, 1:50, Abcam, Cambridge, MA, www.abcam.com). Sections were washed and bound antibody detected using the Immunopure ABC peroxidase staining kit as per the manufacturer's recommendations (Vector Laboratories, Burlingame, CA, www.vectorlabs.com). In order to verify antibody specificity, staining with secondary antibody alone was performed on relevant control tissues (Supporting Information Fig. S6). All stained sections were examined under a Zeiss AxioImager (Z1) upright microscope (Zeiss, Thornwood, NY, www.zeiss.com).

### Statistical Analyses

Quantitative data were obtained in triplicates and reported as mean ± SD, unless otherwise stated. Statistical analyses were performed using Student's *t* test (GraphPad Prism, GraphPad Software, La Jolla, CA, www.graphpad.com), unless otherwise stated, and *p* value < .05 was considered significant.

## Results

### MSC Variability in Colony Formation, Cell‐Size, and Growth

**Figure 1:**
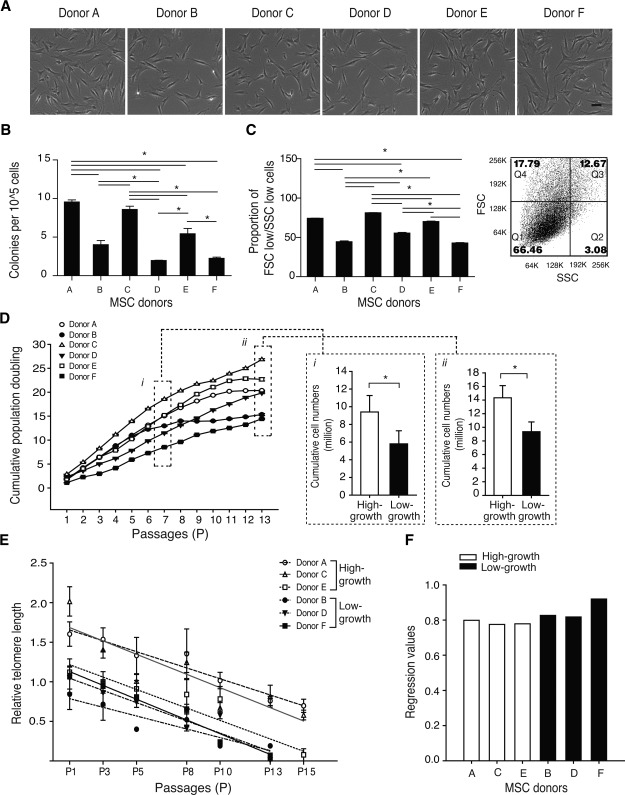
Isolation and growth characteristics of MSCs. **(A):** Phase‐contrast images of MSCs isolated by plastic adherence. All donor MSCs adhered to plastic and were spindle‐shaped in morphology. **(B):** Colony‐forming efficiency of each donor was estimated by enumerating the number of colonies formed per 100,000 bone marrow mononuclear cells plated. *, *p* < .05 ANOVA, with Tukey's multiple comparison test. **(C):** After expanding MSCs for three passages, cells with low forward scatter and low side scatter (Q1) were determined by flow‐cytometry. *, *p* < .05 ANOVA, with Tukey's multiple comparison test. **(D):** Cumulative population doubling from passage 1 (P1) to passage 13 (P13) and cumulative cell number at P7 (i) and P13 (ii) were determined. Difference in growth capacity was observed and donors grouped into those with either high‐ or low‐growth capacity MSCs. *, *p* < .05, Student's *t* test. **(E):** Changes in relative telomere length from P1 to P15; data for Donor D from Samsonraj et. al., (33) was reapplied for comparison purposes. **(F):** Regression values for telomere loss in high‐ and low‐growth MSCs over 15 passages. Each data point from (B) to (F) represents the mean and SD of triplicate experiments. Abbreviations: FSC, forward scatter; MSC, mesenchymal stem cell; SSC, side scatter.

MSCs from multiple human donors are known to vary considerably in their growth parameters. To assess this variability, we examined aspirates from age‐ and sex‐matched donors. Bone marrow mononuclear cells from all donors adhered to tissue culture plastic and gave rise to colonies that readily expanded in culture (Fig. 1A; Supporting Information Fig. S1A). Significant donor variability in CFU‐F efficiency was observed, with donors A, C, and E having greater efficiency (7.4 ± 1.7) than donors B, D, and F (3.2 ± 1.1) (*p* < .001) (Fig. 1B). Nevertheless, all donor MSCs, by virtue of their plastic‐adherence and colony formation, satisfied this aspect of the ISCT criteria.

Donor MSCs were assessed for their cell size. MSC cultures are known to contain a subpopulation of small, round cells that are rapidly self‐renewing (RS), usually identified by flow cytometry as low forward scatter (FSC^lo^) and low side scatter (SSC^lo^) 11, 24, 38. MSCs isolated from donors A, C, and E, with greater colony‐forming ability, had a significantly higher proportion of smaller‐sized cells (74.4%) (FSC^lo^/SSC^lo^ in quadrant 1, Fig. 1C), compared with the proportion of smaller‐sized MSCs from donors B, D, and F (66.4%) (*p* < .001). Donors with higher CFU‐F efficiency also had a higher proportion of small‐sized cells (Figs 1B, 1C).

Long‐term expansion without phenotypic change is a key parameter of MSC quality. We next monitored cell growth for extended periods (8–10 weeks) in culture. Notably, changes in cumulative growth revealed significant differences in the proliferative potential of MSCs from the different donors after seven passages (Fig. 1D). Donor C displayed the greatest growth, with donor F showing the least (Fig. 1D) by passage 13. Cells from donors A, C, and E (high‐growth capacity) achieved 33% more cell numbers over this period compared with donors B, D, and F (low‐growth capacity) (*p* < .05). Thus, we observed that changes in self‐renewal capacity only became evident upon long‐term passaging with MSCs from all donors having similar cumulative growth up to passage 6. Therefore, the high‐growth capacity MSCs seen here were also smaller in size and produced more CFU‐Fs than the low‐growth MSCs.

As telomeres are tightly linked to cell division, we next assessed their status during the serial passaging of MSCs. Analysis from the first passage (P1) until no further proliferation was observed (P13–P15) revealed progressive decreases in relative telomere length (Fig. 1E). The rate of telomere shortening occurred in a linear fashion, as indicated by the negative slope of the curves (Fig. 1E), albeit at different rates as shown by the *r* ^2^ value (Fig. 1F). Regression plots of donors A, C, and E (high‐growth capacity) showed substantially lower regression (*r* ^2^) values than donors B, D, and F (low‐growth capacity), indicating that the high‐growth MSCs reduced their telomeres at slower rates than low‐growth MSCs. In addition, low‐growth MSCs failed to self‐renew after only 13 passages, whereas the high‐growth capacity MSCs continued to proliferate until at least 15 passages. Collectively, our data show that donor MSCs classified as having high‐growth capacity had an increased capacity for self‐renewal, a higher CFU‐F efficiency, and a larger proportion of small‐sized cells.

### Immunophenotypic Profiles of MSCs

**Figure 2:**
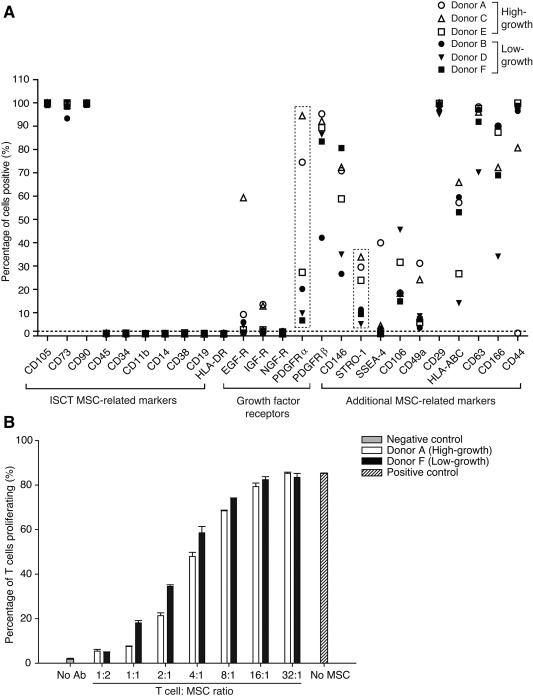
Surface phenotypic profiles and immunosuppression of MSCs. **(A):** Scatter plot of a panel of 25‐cell‐surface biomarkers on P4 MSCs as determined by flow cytometric analysis (fluorescence‐activated cell sorting) from a single experiment; data for Donor E from Samsonraj et. al., (33) was reapplied for comparison purposes. **(B):** Donor MSCs from high‐growth capacity (donor A) and low‐growth capacity (donor F) at P4 were assayed for their immunosuppressive ability in a T‐cell‐based assay. Negative control = absence of MSCs and absence of antibody stimulation of T cells, positive control = absence of MSCs and presence of antibody stimulation of T cells. Each data point in (B) represents the mean and SD of triplicate experiments. Abbreviations: ISCT, International Society for Cellular Therapy; MSC, mesenchymal stem cell.

We next analyzed a set of 25 surface markers including those described by the ISCT 4. Culture‐expanded MSCs from all donors strongly expressed the MSC markers CD105, CD73, and CD90 (greater than 95%) and were negative for the hematopoietic markers CD45, CD34, CD11b, CD14, CD38, CD19, CD31, and HLA‐DR (Fig. 2A); consistent with a preliminary study (33). However, donor variability was seen in the expression of additional markers not included in the ISCT panel. These markers included growth factor receptors and other MSC‐related markers that were selected based on recent reports highlighting their ability to identify MSCs 22, 39. Of the additional markers, PDGFR‐α and STRO‐1 were differentially expressed on the surface of high‐ versus low‐growth capacity MSCs, with high‐growth MSCs having the highest expression levels. We also noted that CD146 was differentially expressed with high‐growth MSCs having the highest expression, with the exception of low‐growth MSCs from donor F. In a similar manner, CD49a was also most highly expressed in high‐growth MSCs, with the exception of donor E. Collectively these data highlight the utility of several additional biomarkers that may be used to further expand upon the standard ISCT panel.

**Figure 3:**
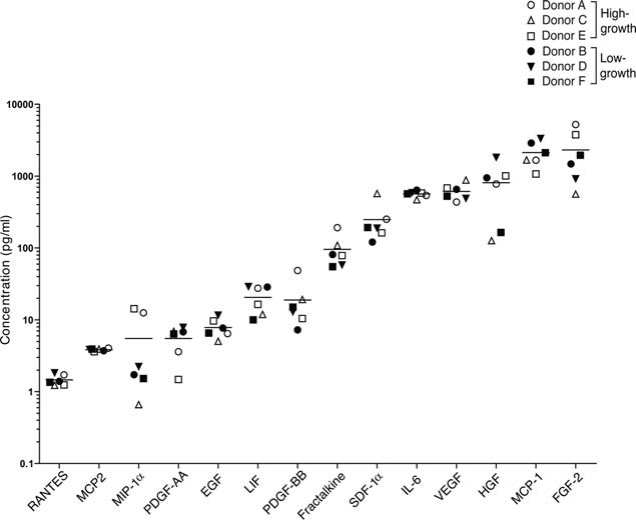
Growth factor and cytokine secretion profiles of mesenchymal stem cells. Cells at P4 were grown in basal media for 4 days and secreted proteins assessed by multiplex immunoassay. Results are expressed in logarithmic concentration as mean of duplicate experiments. Data from the duplicate experiments are presented in Supporting Information Table S6.

### Immunosuppressive Ability of MSCs

Because MSCs are known to suppress T‐cell proliferation 6, 40, we sought to determine whether this functional behavior varied between donors with high‐ and low‐growth capacity MSCs. For this assay, we chose one donor with high‐growth capacity MSCs (donor A) and one donor with low‐growth capacity MSCs (donor F), based on the availability of sufficient cells for the assay. Analysis of T‐cell proliferation by CFDA‐based flow cytometry showed that proliferation under antibody stimulation was suppressed by MSCs in a dose‐dependent manner, irrespective of the donor (Fig. 2B). At a T cell/MSC ratio of 32:1, no suppression was observed with either MSC donor, rather the level of T‐cell proliferation was similar to that observed for antibody‐stimulated T cells alone (no MSC treatment). Notably, inhibition was strongest at a T cell/MSC ratio of 1:2 with only 5.19% of the cultured T cells proliferating (Supporting Information Table S5). At this T cell/MSC ratio, MSCs from both donors were equally able to inhibit T‐cell proliferation (Fig. 2B; Supporting Information Fig. S2). Collectively, our data show that the two donor MSCs are immunosuppressive, independent of their growth status. These results align with a previous study from our lab that demonstrates that the immunomodulation capacity of the MSCs was not diminished, irrespective of their growth capacities 41.

### Secretion Profiles of Growth Factors and Cytokines by MSCs

Some of the regenerative capacity of MSCs has been attributed to the factors they secrete when administered to treat disease or injury (see review 29). To assess this function, we assayed a set of 14 growth factors and cytokines previously associated with key MSC functions 29 such as immunomodulation (HGF and leukemia inhibitory factor (LIF)), chemoattraction (RANTES, SDF‐1α, fractalkine, MIP‐1α, MCP‐1, and MCP‐2), support of progenitor cells (IL‐6, FGF‐2, PDGF‐AA, PDGF‐BB, and EGF), angiogenesis (VEGF_165_, FGF‐2, PDGF‐AA, PDGF‐BB, and EGF), antiscarring (HGF and FGF‐2), and antiapoptosis (FGF‐2, HGF, and VEGF_165_). All donor MSCs secreted detectable amounts of the various factors, albeit at varying amounts (Fig. 3; Supporting Information Table S3). Data from duplicate experiments for each donor show similar secretion levels for each of the factors tested and are presented as a mean value (Fig. 3; Supporting Information Tables S3, S6). Also, for each of these factors, no difference was observed between the high‐ or low‐growth capacity groups (Supporting Information Fig. S3). However, secretion levels varied from <10 pg/ml for RANTES, MCP‐2, MIP‐1α, PDGF‐AA, and EGF; 10–100 pg/ml for LIF, PDGF‐BB, and fractalkine; 100–1,000 pg/ml for SDF‐1α, IL‐6, VEGF_165_, and HGF; and >1,000 pg/ml for MCP‐1 and FGF‐2 (Supporting Information Table S3). It is to be noted that the analysis of growth factor and cytokine secretion were performed using cells at passage 4 at which stage the levels of secretion between the two groups were not significantly different. At this passage, we did not observe significant differences in the growth capacity between the two groups, as can be seen from their cumulative cells numbers (*p* value = .144), population doubling, PD (*p* value = .337), and cumulative PD (*p* value = .166) (Supporting Information Fig. S1C).

### Gene Expression Analysis

**Figure 4:**
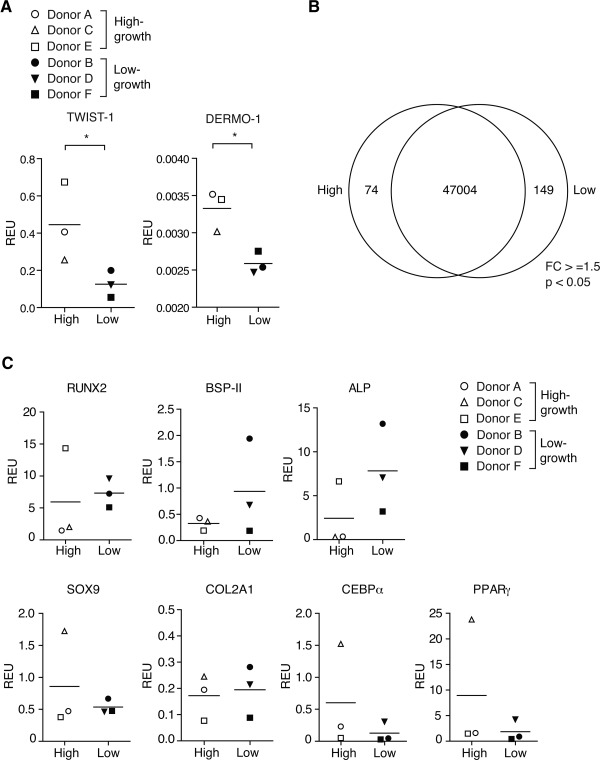
Gene expression analysis. **(A):** Quantitative PCR analysis of the mesoderm‐related mRNA transcripts *TWIST‐1* and *DERMO‐1* in high‐ and low‐growth capacity mesenchymal stem cells (MSCs) at P4. **(B):** Venn diagram showing global gene expression analysis of high‐and low‐growth capacity MSCs determined by microarray analysis at P4. Only transcripts with a FC ≥1.5 and *p* value <.05 were included. **(C):** Quantitative PCR analysis of lineage‐specific markers in P4 cells cultured for 14 days under noninduced conditions. Graphs are represented as relative expression units compared with β‐actin. Each data point represents the mean of triplicate experiments. Abbreviations: *ALP*, alkaline phosphatase; *BSP II*, bone sialoprotein II; *CEBPα*, CCAAT enhancer binding protein alpha; *Col2A1*, collagen, type II, alpha 1; FC, fold change; *PPARγ*, peroxisome proliferator‐activated receptor gamma; REU, relative expression units; *RUNX2*, Runt‐related transcription factor 2.

To identify differences between the high‐ or low‐growth capacity cells at the mRNA level, qPCR was performed to assess the levels of mesoderm‐related markers *TWIST‐1* and *DERMO‐1*. High‐growth capacity MSCs demonstrated significantly higher (*p* ≤ .05) transcript levels of these markers (Fig. 4A). A global gene expression analysis was next performed on these two groups. A fold change cut‐off of 1.5 and *p* ≤ .05 delineated a small set of transcripts differentially expressed between the two groups, with 74 genes significantly enriched in the high‐growth MSCs and 149 in the low‐growth capacity MSCs (Fig. 4B). The limited size of the distinguishing gene sets indicates that the two groups are largely similar and that the trait variation could be attributed to these genes.

Gene ontology analysis by DAVID functional annotation clustering 34 was performed on the gene lists generated to identify biological processes enriched in the high‐ and low‐ growth MSC groups. Genes regulating cell adhesion and the organization of cytoskeleton and organelles were significantly upregulated (*p* ≤ .05) in high‐growth capacity group (Supporting Information Table S4A), which are indicative of a faster rate of turnover. Adhesion to the matrix and neighboring cells correlates with cell cycle progression 42, 43. Genes enriched in the low‐growth MSCs tend to be cell morphogenetic and related to the development of mesenchyme‐derived organs (Supporting Information Table S4B).

In order to investigate the upregulation/downregulation of genes involved in MSC differentiation, we assayed for the transcript levels of *RUNX2*, *BSP‐II*, *ALP*, *COL2A1*, *SOX9*, *CEBPα*, and *PPARγ* under noninduced conditions by qPCR (Fig. 4C). Individual donors demonstrated some variability in the baseline expression of these genes; however, no difference in these trilineage differentiation markers was observed between the two groups.

### Multilineage Differentiation Ability

**Figure 5:**
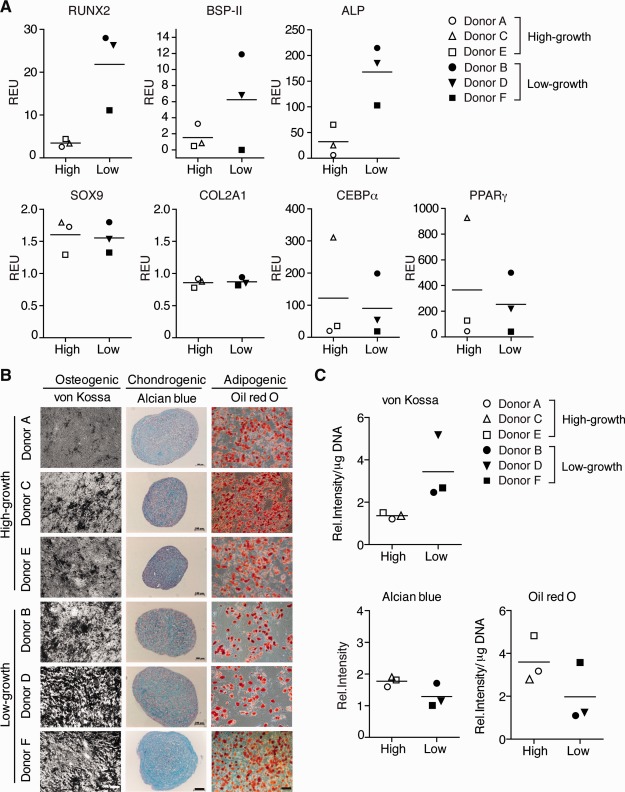
Multilineage differentiation. Multilineage ability was determined in P4 mesenchymal stem cells. **(A):** Quantitative PCR analysis of osteogenic markers *RUNX2*, *BSP‐II*, and *ALP*, chondrogenic markers *COL2A1* and *SOX9*, and adipogenic markers *CEBPα* and *PPARγ* in cells cultured for 14 days under the respective lineage induction conditions. Scatterplots are represented as relative expression units compared with β*‐ACTIN*. **(B):** Representative images with von Kossa, oil red O, and alcian blue staining. Scale bar = 10 µm. **(C):** Quantification of stains by image analysis normalized to DNA content. Each data point represents the mean of triplicate experiments. Abbreviations: *ALP*, alkaline phosphatase; *BSP II*, bone sialoprotein II; *CEBPα*, CCAAT enhancer binding protein alpha; *Col2A1*, collagen, type II, alpha 1; *PPARγ*, peroxisome proliferator‐activated receptor gamma; REU, relative expression units; *RUNX2*, Runt‐related transcription factor 2.

To assess the multipotency of the MSCs from the various donors, cells were induced to differentiate down the osteogenic, adipogenic, and chondrogenic lineages by culturing them with defined media components and culture conditions. All donor MSCs demonstrated trilineage differentiation ability (Fig. 5, Supporting Information Fig. S4). With the exception of *RUNX2*, all mRNA transcripts assessed were upregulated when MSCs were exposed to the various lineage‐induction conditions (Figs. 4C, 5A). Moreover, stains for bone (von Kossa), cartilage (alcian blue), and fat (oil red O) further highlight similarities in the in vitro differentiation capacity of high‐ and low‐growth MSCs (Fig. 5B, 5C) and suggests this criteria is not sufficient to distinguish between these two groups of cells.

### In Vivo Ectopic Bone‐Forming Efficacy of MSCs

**Figure 6:**
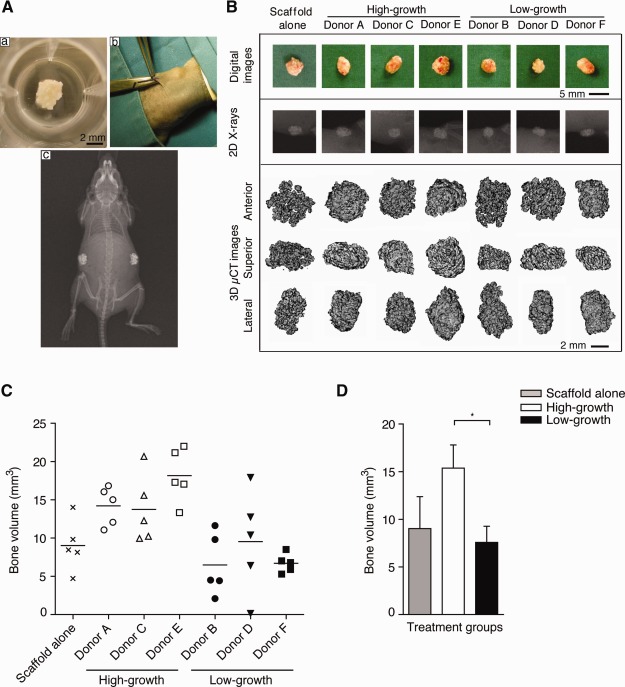
Ectopic bone formation. **(Aa, Ab):** Representative images of mesenchymal stem cells (MSCs)‐loaded scaffolds implanted at subcutaneous sites. **(Ac):** X‐ray showing two MSC‐loaded implants 8 weeks after implantation. **(B):** Panel showing representative digital images, 2D x‐rays, and µCT reconstructions of implants after 8 weeks. **(C):** Scatter plot showing new bone volume generated by MSC‐loaded scaffolds from each donor. Scaffold without cells (scaffold alone) was included as control. **(D):** Bar graph comparing volume of new bone formation between high‐ and low‐growth capacity MSC‐loaded scaffolds; *, *p* < .05, Student's *t* test. Each data point in (C) represents a single experiment and each data point in (D) represents the mean and SD from the data in the scatter plot (C). Scaffold alone *n* = 5, high‐growth capacity MSC‐loaded scaffold *n* = 15, low‐growth capacity MSC‐loaded scaffold *n* = 15.

**Figure 7:**
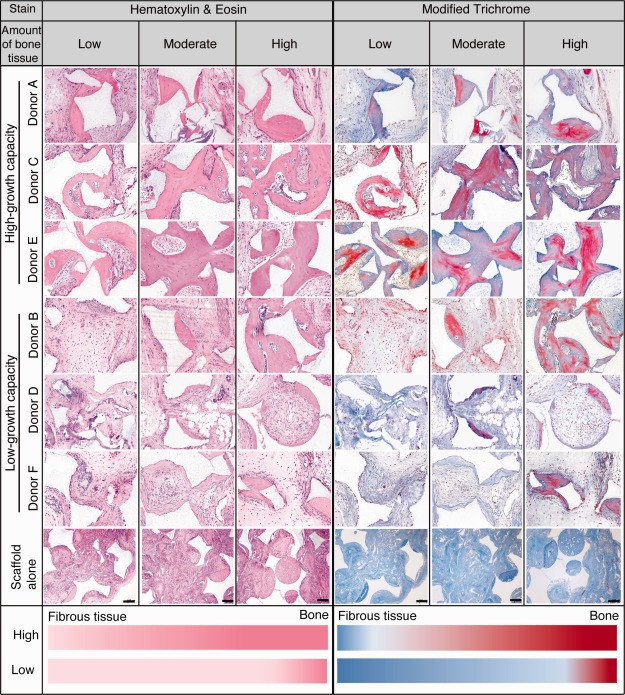
Histological analysis of ectopic bone formation. Representative images of implant sections stained with hematoxylin & eosin (H&E) and modified trichrome. Reading each panel from left to right are representative images of areas containing low (left) to high (right) bone formation. Increased variability in the amount of bone tissue is observed for implants containing low‐growth capacity mesenchymal stem cells when compared with implants containing high‐growth capacity cells. Color gradient at the bottom of the figure indicates relative changes in tissue phenotype. H&E panel: light pink represents fibrous tissue and dark pink bone tissue. Modified trichrome panel: pale blue represents fibrous tissue and bright red, mineralized bone tissue. Scale bar = 50 µm.

The in vitro characterization of MSCs was followed by a functional in vivo assay to determine the ability of high‐ and low‐growth capacity MSCs to contribute to the formation of ectopic bone in mice over an 8‐week period (Fig. 6A). MSCs from all donors demonstrated a capacity to form ectopic bone, albeit to varying extents. The scaffold, MASTERGRAFT Matrix, with no MSCs produced some bone, in keeping with the osteoconductive property of this scaffold. However, scaffold seeded with high‐growth capacity MSCs exhibited a significantly increased ability to form ectopic bone (*p* < .05) compared with both scaffold alone or scaffold seeded with low‐growth capacity MSCs, as determined by µCT analysis (Figs 6B–6D). Notably, no difference in the ability to form ectopic bone was observed between scaffold alone and scaffold seeded with low‐growth MSCs. Histological sections taken through the implants stained with both H&E and modified trichrome permitted a more detailed assessment of tissue morphology as well as the extent of ectopic bone formation versus fibrous tissue. Our data show that scaffold seeded with high‐growth MSCs (donors A, C, and E) readily produced bone tissue that also contained increased amounts of mineral (highlighted by the increased red staining in the accompanying modified trichrome sections) (Fig. 7). Further microscopic examination of each implant highlighted areas that contained relatively low amounts of bone tissue as well as areas with moderate and high amounts of bone tissue. However, of particular note was the observation that scaffold seeded with high‐growth capacity MSCs contained more bone tissue throughout the implant, even in areas described as having low amounts of bone tissue, when compared with both scaffold alone or scaffold seeded with low‐growth capacity MSCs that contained increased amounts of fibrous tissue, which has been represented as a color gradient bar (Fig. 7). Based on morphological assessment of all the implant sections from different donors, we identify that the lowest amount of bone tissue formed by high‐growth capacity MSCs was higher than the average amount produced by low‐growth capacity donors. We also evaluated the species origin of the new bone tissue formed by staining with antibodies against human and mouse osteocalcin. Our results show that the bone formed was of a mixture of both human and mouse tissue (Supporting Information Figs. S5, S6). Scaffolds seeded with high‐growth capacity MSCs showed relatively higher levels of human osteocalcin staining compared to low‐growth MSCs.

Taking into consideration all the results above, we identified the following in vitro parameters that positively correlate with in vivo bone formation: cell size (*r* ^2^ value = 0.74), cumulative cell number (*r* ^2^ value = 0.65), STRO‐1 expression (*r* ^2^ value = 0.55), mRNA level of TWIST‐1 (*r* ^2^ value = 0.8), and mRNA level of DERMO‐1 (*r* ^2^ value = 0.72). The regression plots are provided as Supporting Information Figure S7.

## Discussion

To date, no systematic correlation between the properties of MSCs in vitro and their in vivo efficacy has been demonstrated, primarily because there is no universally accepted in vitro method for predicting the therapeutic potency of MSCs. A key study attempting to provide clinically relevant potency assays demonstrated that the doubling time of MSCs correlates with in vivo bone formation 44; here, we have extended that concept to include other notable in vitro parameters that may help to better define MSC quality, such as colony‐forming efficiency 9, cell size 24, immunosuppression 40, and cytokine and growth factor secretion 29. Indeed, a recent review by Prockop et al. 8 highlighted the need for the benchmarking of MSCs against an established reference MSC line. In this study, we have used a subcutaneous ectopic bone formation assay for assessing MSC potency, the reason being ectopic models offer unique advantages over orthotopic (bone) environments, including a relative lack of bone cytokine stimulation and cell‐to‐cell interaction with endogenous (host) bone‐forming cells 45. This allows for relatively controlled in vivo experimental bone formation and better analysis of the new bone formed. Although this study does not address the potential potency of these cells for other clinical applications (such as cardiac and inflammatory conditions), our work does offer new insight into selecting potent MSCs using a simple ectopic bone formation model. The data obtained from this study should facilitate future evaluation of the varying growth capacity of MSCs and the relationship to other clinical applications that can be assessed using relevant preclinical models.

By comparing our in vitro findings with in vivo bone formation ability, we conclude that MSCs with higher CFU‐F efficiencies, a larger proportion of small‐sized cells, and enhanced growth capacity were able to form increased amounts of bone at ectopic sites. Another important outcome from this study was the need to include additional cell‐surface biomarkers in the assessment for the identification of MSC, thus highlighting that the ISCT panel of surface markers did not adequately predict the in vivo potency of MSCs. Notably, in this study, the use of additional surface markers such as STRO‐1 and PDGFR‐α was able to distinguish between MSCs with high versus low ectopic bone‐forming ability, and thus may help to identify more potent MSCs. Importantly, the expression of STRO‐1 on bone marrow‐derived MSCs has been extensively studied 17, 18, 46 and our results are supportive of previous investigations showing STRO‐1+ cells having improved growth and colony‐formation ability. More recent studies have indicated that PDGFR‐α is expressed by nestin+ MSCs 47, and PDGFR signaling is a key regulator of MSC potency 48. Our finding that the high‐growth capacity MSCs (with increased bone‐forming ability) also possess higher expression levels of STRO‐1 and PDGFR‐α, further corroborate these investigations.

Another feature of MSCs pertains to their immunomodulatory activities. These cells appear to have potent immune regulatory functions that make them good therapeutic candidates for tissue injury and/or inflammation 40. It is evident from various studies that the mechanisms underlying MSC‐mediated immunosuppression are contact‐dependent and ‐independent 49, 50, 51 which include suppression of T cells 51, inhibition of dendritic cell differentiation and B‐cell proliferation 52. Our data support these findings and show that stimulated T‐cell proliferation can be suppressed by MSCs, so highlighting the importance of including MSC‐mediated immunosuppression in an assessment of MSC quality. For the assessment of MSC immunosuppression, we used purified T cells as effector cells rather than unselected PBMCs that provide less reproducible results owing to the indirect effect of monocytes. Our method of studying immunosuppression is in agreement with the recent suggestions provided by the ISCT for better evaluating the relative functional potency of MSCs 31.

In addition to phenotypic and functional assays of MSCs in this study, a global gene expression analysis was performed. Results revealed the enrichment of proliferation‐associated processes in high‐growth MSCs and maturation‐related processes in low‐growth MSCs, which together suggest a physiological basis that may underpin their phenotypic divergence in vitro. As the MSCs were derived and propagated from donors according to standardized procedures, differences in the physiology of the cells might be due to an underlying variation in their transcriptome. Also, increased expression of *TWIST‐1* and *DERMO‐1*, mRNA transcripts shown by Psaltis et al. 46 to be enriched in STRO‐1+ MSCs and known to be crucial for MSC growth and development, further attests to the possible inclusion of these markers in MSC assessment.

There is a growing acceptance that the tissue regeneration capacity of MSCs is driven, in part, by the factors they secrete when transplanted to sites of injury or disease 5. However, to the best of our knowledge, donor to donor variability has not been reported in the levels of key regenerative growth factors and cytokines secreted by MSCs. Among the many growth factors regulating bone metabolism, FGF‐2 and VEGF are recognized as autocrine or paracrine factors crucial for osteoblasts differentiation 53, 54 and angiogenesis 55, respectively. Analysis of the expression levels in MSCs of growth factors and cytokines important in tissue repair showed little variability between donors, irrespective of grouping based on growth capacity. Despite this finding, increased amounts of ectopic bone were formed by high‐growth capacity MSCs compared to low‐growth capacity MSCs. Because the high‐growth capacity MSCs lose telomeres at slower rates, it may be possible that they are able to undergo more divisions before senescence, thus providing an advantage in sustaining the production of key cytokines for longer periods of time (after transplantation), rather than the absolute amounts they produce. Furthermore, the bone formed was a mixture of human (donor) and mouse (host) tissue suggesting that the transplanted MSCs may have differentiated into osteogenic cells that deposit human tissue while also being able to stimulate the formation of host (mouse) bone. However, the underlying mechanism driving this host response remains to be elucidated, particularly because this study failed to show any difference in the in vitro secretory levels of key osteogenic factors with passage 4 MSCs. As growth differences became evident only after passage 7, it would be interesting to compare cytokine secretion and their autocrine effects on the MSCs at later passages. It is possible that MSCs when transplanted secrete levels of reparative factors that may not be correlative with their in vitro secretions, an area that needs further investigation.

Although both groups of MSCs secreted similar levels of growth factors and cytokines and showed comparable capacities for osteogenic differentiation in vitro, they produced varying amounts of ectopic bone in vivo. This could be because the high‐growth MSCs were able to survive longer and have sustained growth because of relatively longer telomeres and a higher proportion of clonogenic small‐sized cells. The deposition of bone in the scaffold suggests that the capacity to deposit a mineralized matrix is spatially controlled, and that the most adaptable MSC to achieve this may be the high‐growth capacity cells. That the standard in vitro osteogenic assay yields a poor prognosis is in accord with a previous study from our group and others showing that ex vivo matrix mineralization assays lack specificity, and shows little or no concordance with true bone formation 15, 56. We have also shown recently that culture media containing a particular heparan sulfate glycosaminoglycan can be used to enrich for a population of potent MSCs with increased self‐renewal, longer telomeres, and enhanced survival and bone reparative properties when transplanted in vivo 41. Of particular note, data from this study show that high‐growth capacity cells shorten their telomeres at slower rates compared with low‐growth capacity MSCs and continued to proliferate for longer periods, suggesting that they may also have increased survival when transplanted in vivo. Support for this is provided by the finding that high‐growth capacity MSCs stimulated greater amounts of ectopic bone formation that was a mixture of human and mouse origin, thus suggesting that the transplanted cells may differentiate down the osteogenic lineage and deposit bone‐like tissue as well as stimulated the host to deposit new bone‐like tissue. Similarly, decellularized extracellular matrix (ECM) substrates have proven useful for the expansion of multipotent adult stem cells that are smaller in size and more homogenous 57, thus highlighting the role of the culture microenvironment in the maintenance of MSC potency.

Despite the immense amount of work done over the last decade, MSCs are still a relatively poorly understood, heterogenous mixture of cells with unpredictable properties. As pointed out by Mendicino et al. in their recent review 3, there is bewildering diversity in how sponsors have defined, manufactured, and described MSCs in their regulatory submissions to the U.S. FDA, not only in terms of tissue sourcing, but also in methods of in vitro propagation, cell‐surface marker expression, and product manufacturing. Survey of FDA submissions shows that seven cell‐surface markers are routinely used for MSC‐based product IND submissions (CD105, CD73, CD90, CD45, CD34, CD14, and HLA class II), which is consistent with the marker set specified by the ISCT 4. However, it is clear that this marker set is far from definitive, and encompasses a vast majority of cells without true, “stem‐like” qualities; there is still an open question as to which particular set of markers truly describes this heterogeneous cell class. Here, we advocate the combined use of preclinical animal studies coupled with a broader set of in vitro characteristics that may be used to define MSC potency. More work of this kind is needed to provide clinicians with MSCs of the highest quality for therapeutic use.

## Conclusion

We have identified the MSC characteristics of cell size and in vitro growth capacity, together with expression of STRO‐1, TWIST‐1 and DERMO‐1 as being positively correlated with ectopic bone‐forming ability of implanted MSCs. We thus advocate the use of such additional criteria when assessing MSC quality before applying them as a cellular therapy.

## Author Contributions

R.M.S.: conception and design, collection and assembly of data, data analysis and interpretation, and manuscript writing; B.R.: collection of data and data analysis and interpretation; M.R.: data analysis and interpretation and manuscript review; J.H.H.: financial support, provision of study material, and manuscript review; K.J.P.: experiment design, collection and assembly of data, data interpretation, and manuscript review; O.R.: provision of study material, data analysis and interpretation, and manuscript review; P.S.: collection and assembly of data, data analysis and interpretation, and manuscript writing; L.W.S.: conception and design, provision of study material, data analysis and interpretation, and manuscript review; V.N.: conception and design, financial support, data analysis and interpretation, and manuscript writing; S.M.C.: conception and design, financial support, provision of study material, data analysis and interpretation, manuscript writing, and final approval of manuscript.

## Disclosure of Potential Conflicts of Interest

The authors indicate no potential conflicts of interest.

## Supporting information

Supplementary Information Figure 1 R2

Supplementary Information Figure 2

Supplementary Information Figure 3

Supplementary Information Figure 4

Supplementary Information Figure 5

Supplementary Information Figure 6

Supplementary Information Figure 7

Supplementary Information Data

Supplementary Information Legends
