# Supplementary figures and images for: Establishing Criteria for Human Mesenchymal Stem Cell Potency
Source: Stem Cells. 2015 May 21;33(6):1878–91. doi: 10.1002/stem.1982 (PMC5363381; doi:10.1002/stem.1982)

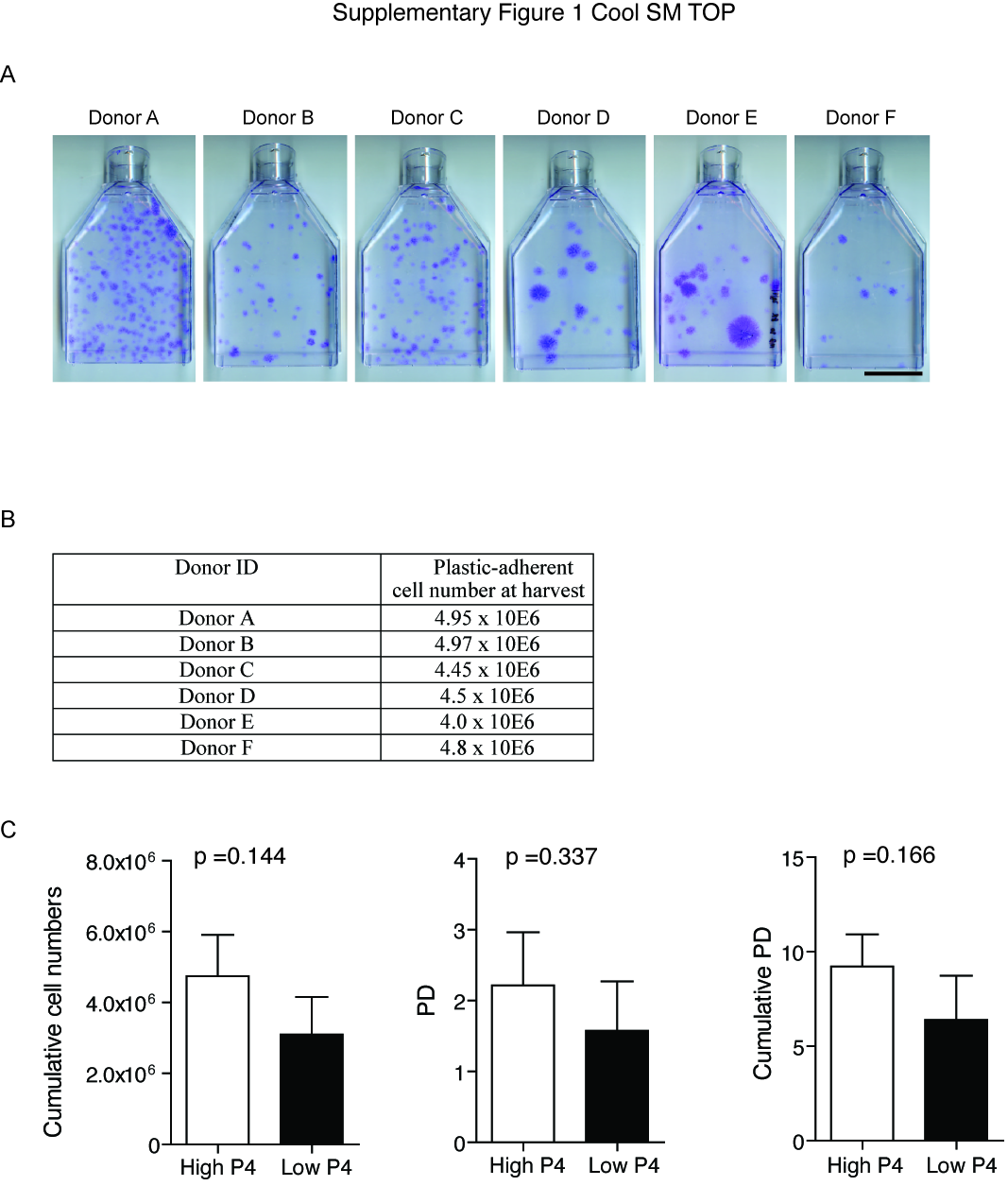

Supplement: Supplementary file 1 — Supplementary Information Figure 1 R2 [file STEM-33-1878-s001.tif]

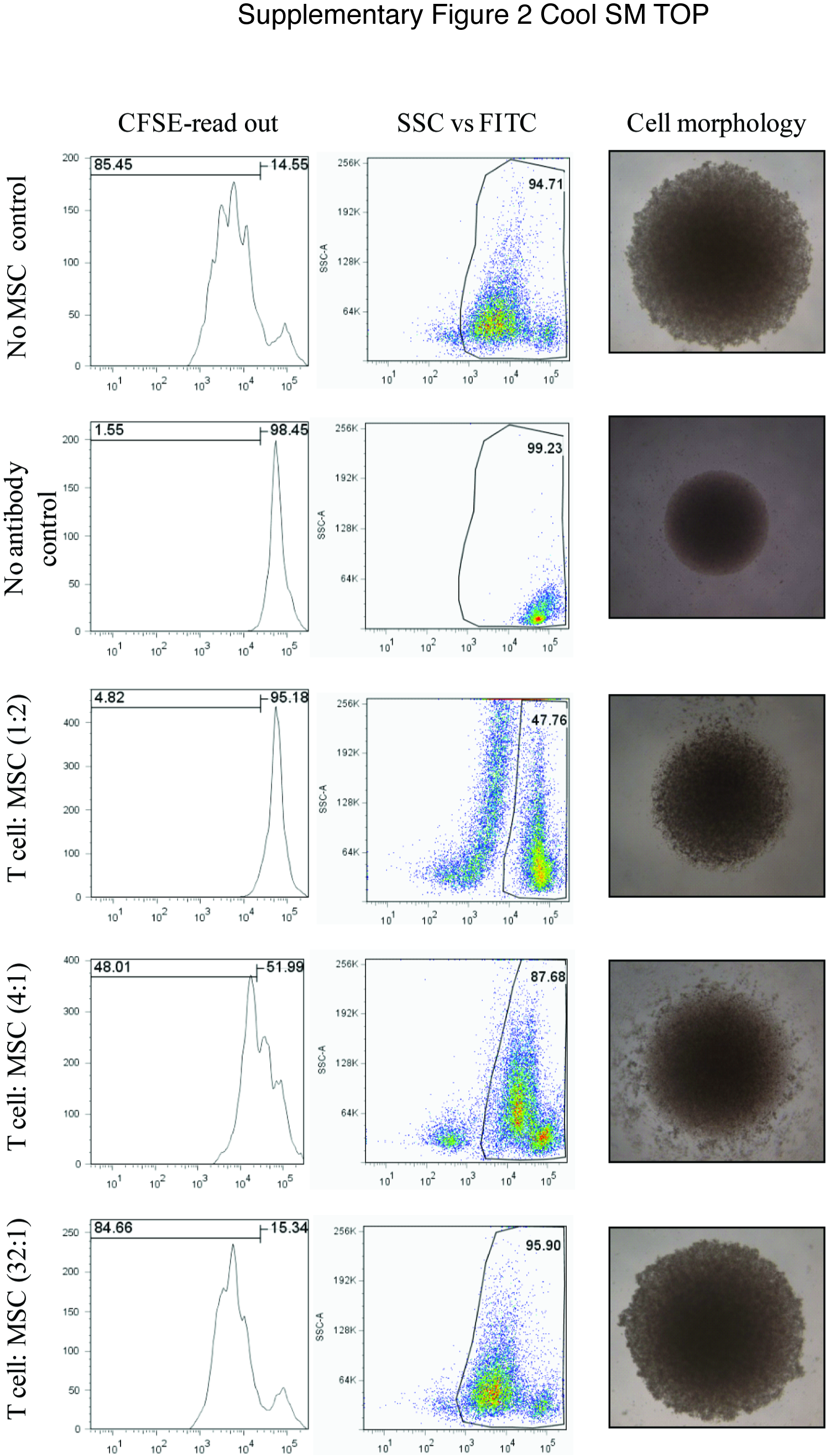

Supplement: Supplementary file 2 — Supplementary Information Figure 2 [file STEM-33-1878-s002.tif]

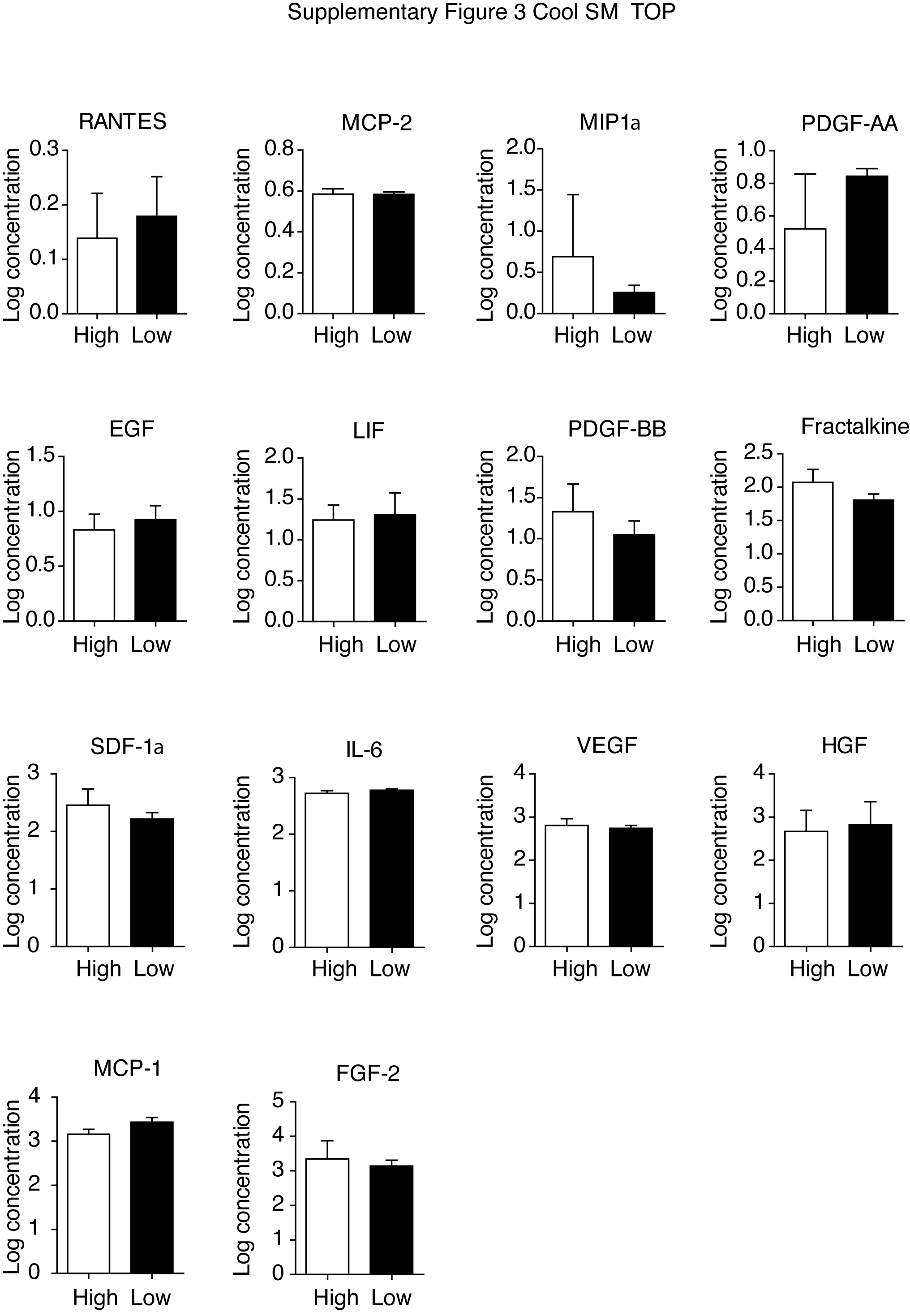

Supplement: Supplementary file 3 — Supplementary Information Figure 3 [file STEM-33-1878-s003.tif]

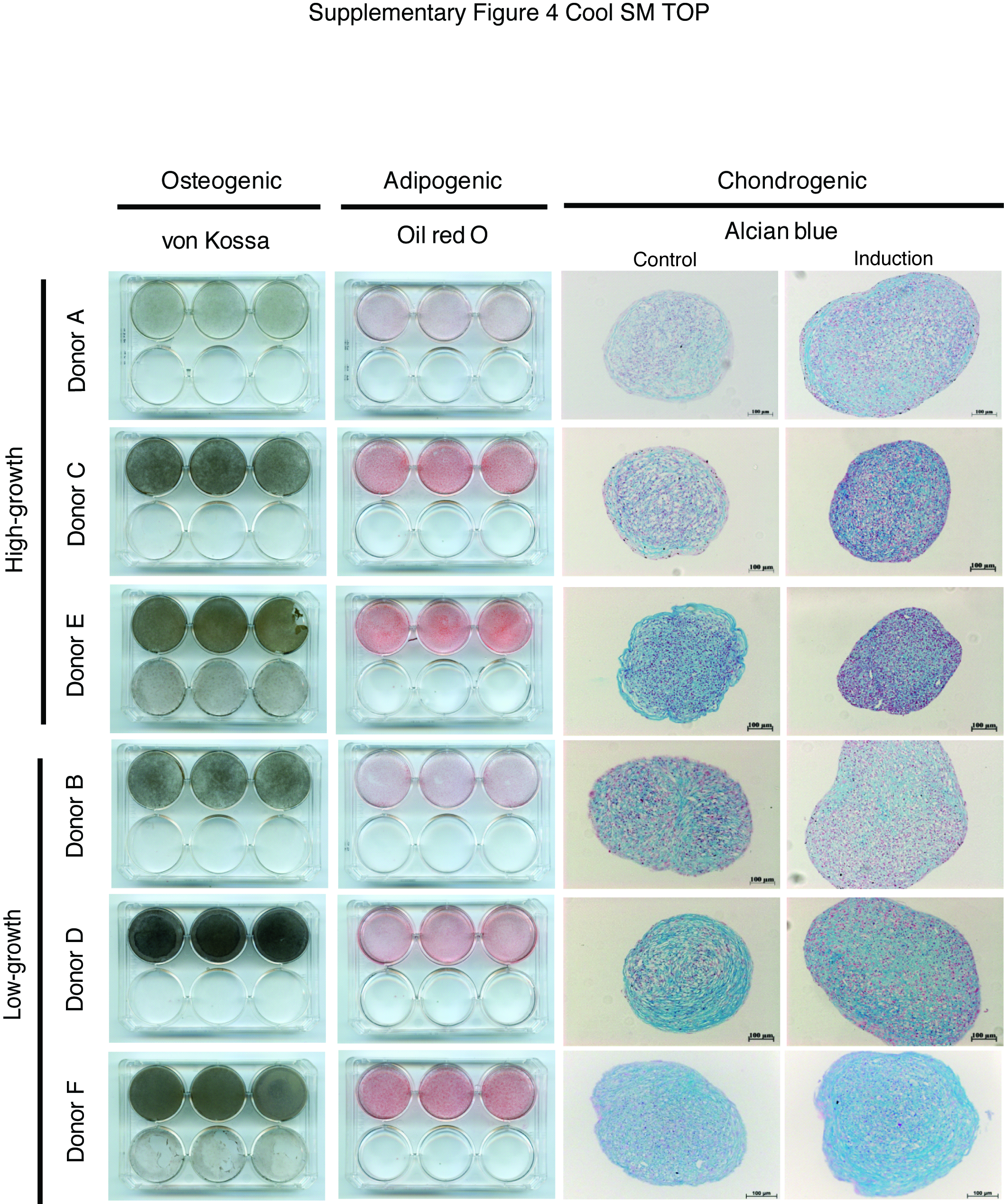

Supplement: Supplementary file 4 — Supplementary Information Figure 4 [file STEM-33-1878-s004.tif]

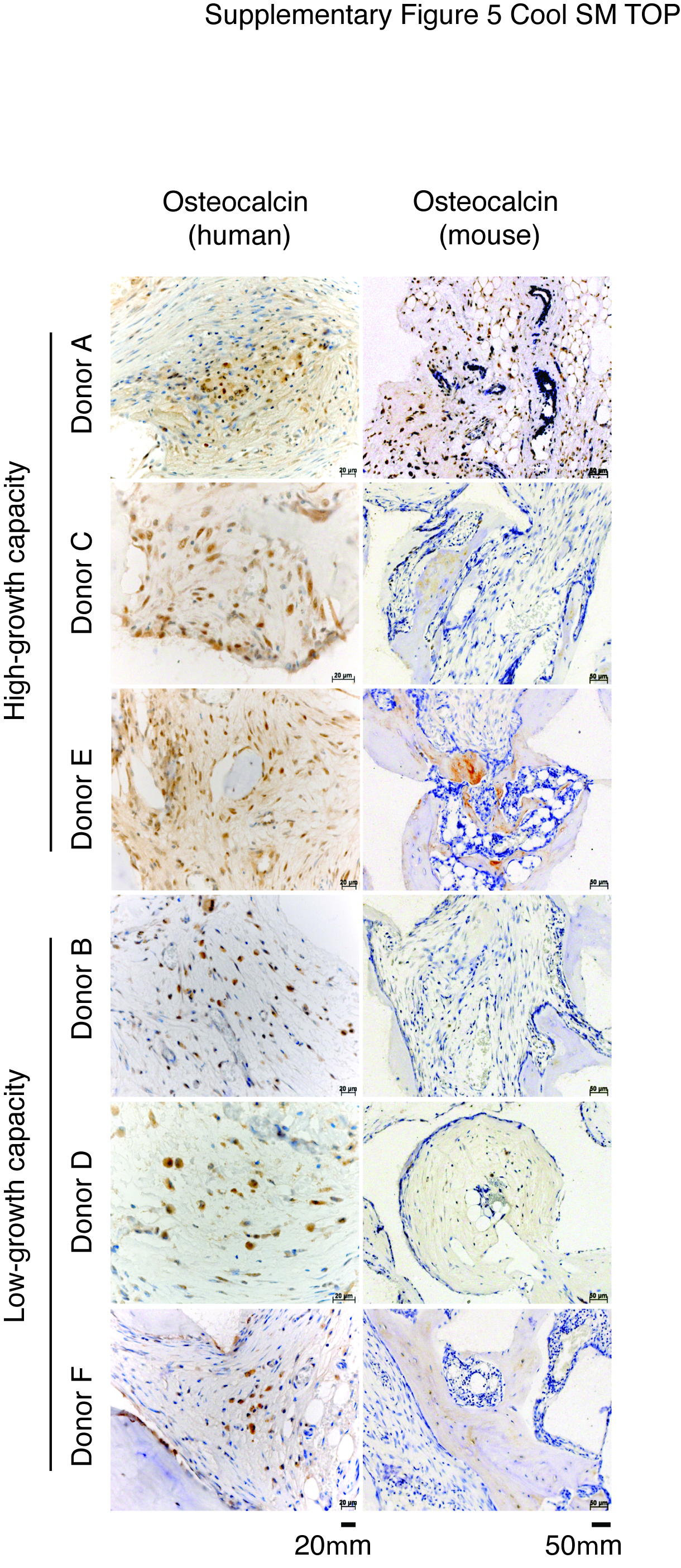

Supplement: Supplementary file 5 — Supplementary Information Figure 5 [file STEM-33-1878-s005.tif]

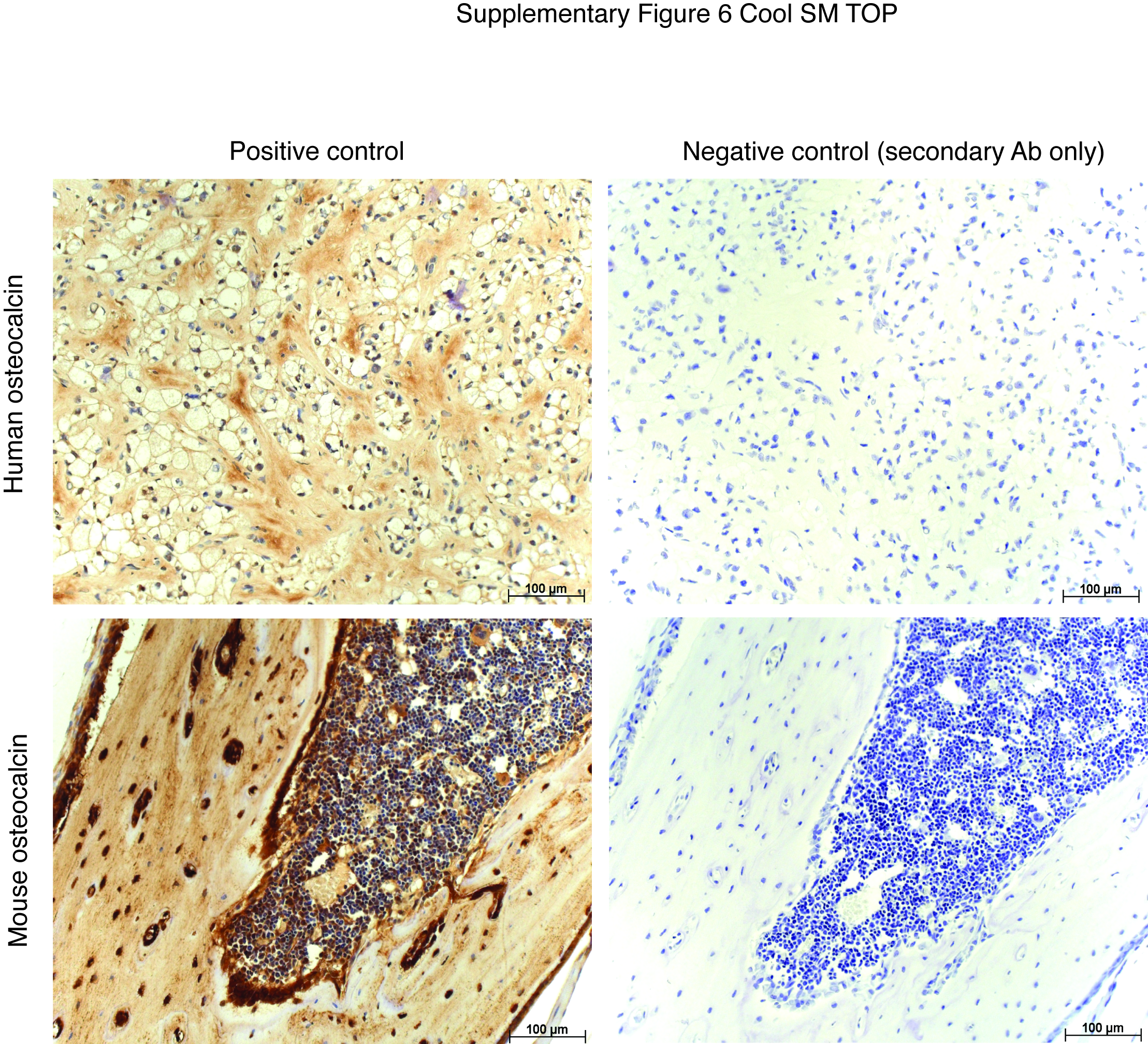

Supplement: Supplementary file 6 — Supplementary Information Figure 6 [file STEM-33-1878-s006.tif]

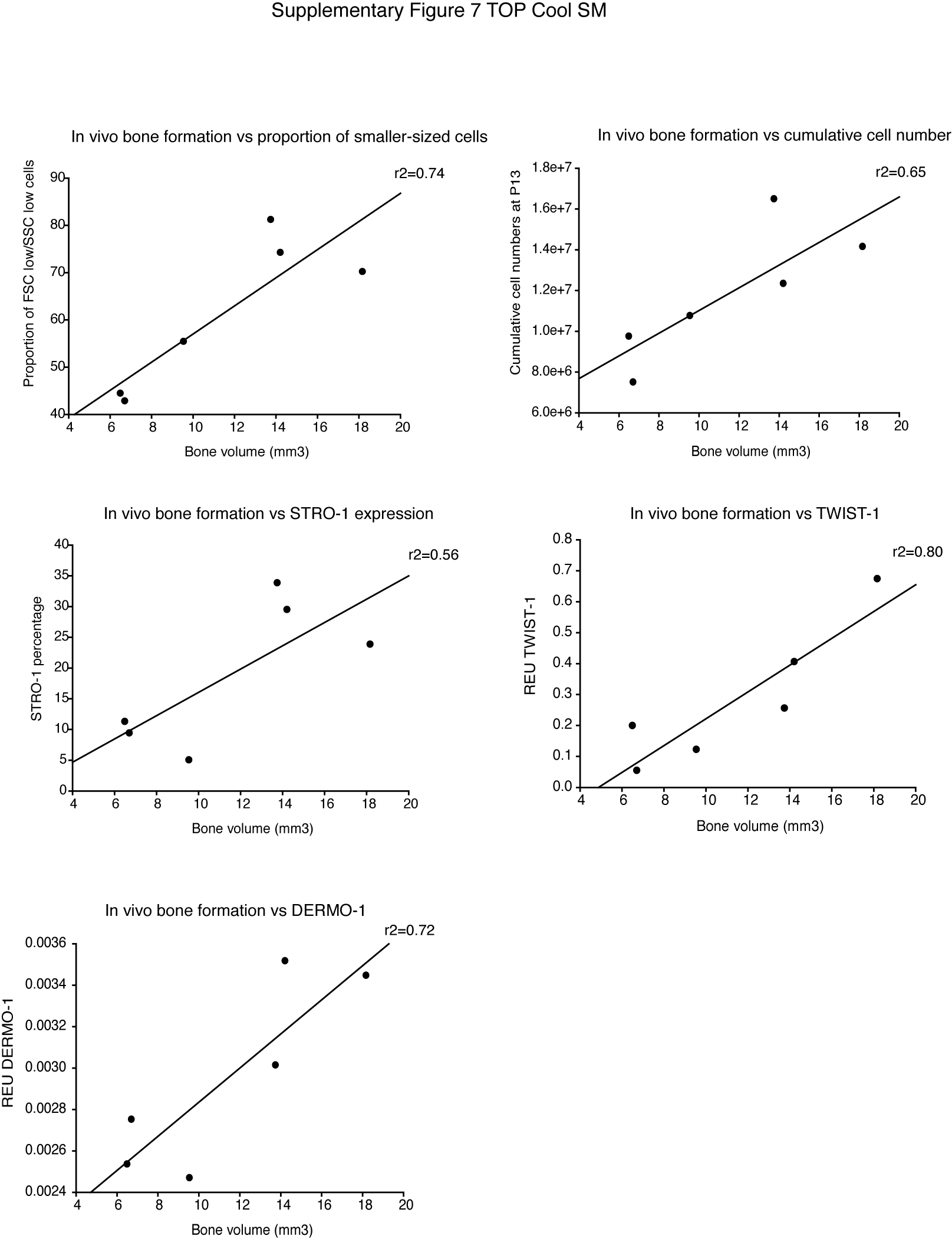

Supplement: Supplementary file 7 — Supplementary Information Figure 7 [file STEM-33-1878-s007.tif]
