# Supplementary material for: Establishing Criteria for Human Mesenchymal Stem Cell Potency
Source: Stem Cells. 2015 May 21;33(6):1878–91. doi: 10.1002/stem.1982 (PMC5363381; doi:10.1002/stem.1982)
Supplement: Supplementary file 9 — Supplementary Information Legends [file STEM-33-1878-s009.doc]

**Supplementary Figure 1**. **Colony formation assay**. **A.** Colonies of MSCs formed when plating bone marrow mononuclear cells from 6 different donors. Scale bar = 40 mm. **B**. Quantification of plastic-adherent cells harvested upon isolation from the bone marrow mononuclear cells of six donors. **C**. Comparison of cumulative cell numbers, population doubling (PD), and cumulative population doubling (CPD) between high- and low-growth capacity cells at passage 4. No significant differences were observed at passage 4, *p* >0.05.

**Supplementary Figure 2. Representative immunosuppressive assays.** T cells and MSCs from a representative high-growth capacity donor (donor A) or a representative low-growth capacity donor (donor F) were co-cultured in varying proportions of T cells: MSCs. The percentage of T cells proliferating can be derived from the CFSE-read out (read by the FITC channel) where peaks show successive cell divisions. The scatter plots (SSC vs FITC) indicate the percentage of cells positive for FITC. Differences in the size of the proliferating T cell colonies are visualized by the T cell micrographs of individual wells of a 96-well plate. Positive controls (no MSCs) and negative control (no antibody) are also included.

**Supplementary Figure 3. Cytokine and growth factor secretions by MSCs.** Bar graph showing average secretion levels (log transformation of concentration) of cytokines and growth factors between high- and low-growth capacity MSCs. Data points are means of duplicate experiments for each donor with data from the duplicate experiments presented in Suppl. Table 6.

**Supplementary Figure 4**. **Multilineage differentiation.** Scanned images of 6-well plates showing differences between the staining intensity of induced (top wells) versus uninduced control cultures (bottom wells) stained by von Kossa or Oil red O and pellet section stained with Alcian blue.

**Supplementary Figure 5. Immunohistochemistry.** Sections of implants from each donor showing staining for anti-human osteocalcin (left panel, scale bar 20 µm) and anti-mouse osteocalcin (right panel, scale bar 50 µm).

**Supplementary Figure 6. Immunohistochemistry of tissue control samples.** Sections of human hepatocellular carcinoma tissue stained with anti-human osteocalcin and its corresponding secondary antibody alone to show the specificity of the antibody used (top). Sections of mouse femur tissue stained with anti-mouse osteocalcin and its corresponding secondary antibody alone to show the specificity of the antibody used (bottom). Scale bar: 100 µm.

**Supplementary Figure 7: Regression plots of *in vivo* bone formation vs *in vitro* parameters.** Linear regression plots showing the correlation between *in vivo* ectopic bone volume and *in vitro* parameters such as cell-size, cumulative cell number, STRO-1 expression, mRNA levels of *TWIST-1* and *DERMO-1*.
